# Vav1 mutations identified in human cancers give rise to different oncogenic phenotypes

**DOI:** 10.1038/s41389-018-0091-1

**Published:** 2018-10-08

**Authors:** Batel Shalom, Marganit Farago, Eli Pikarsky, Shulamit Katzav

**Affiliations:** 1 Developmental Biology and Cancer Research, IMRIC, Hebrew University-Hadassah Medical School, Jerusalem, Israel; 2 Department of Immunology and Cancer Research, IMRIC, Hebrew University-Hadassah Medical School, Jerusalem, Israel; 3 Department of Pathology, IMRIC, Hebrew University-Hadassah Medical School, Jerusalem, Israel

## Abstract

Vav1 is physiologically active as a GDP/GTP nucleotide exchange factor (GEF) in the hematopoietic system. Overexpression of Vav1 in multiple tumor types is known to enhance oncogenicity, yet whether or not Vav1 is a bona fide oncogene is still a matter of debate. Although mutations in Vav1 were recently identified in human cancers of various origins, the functional activities of these mutants are not known. We tested the transforming potential of three mutations identified in human lung adenocarcinoma: E59K, D517E, and L801P. Results from several assays indicative of transforming activities such as rate of proliferation, growth in agar, and generation of tumors in NOD/SCID mice clearly indicated that E59K and D517E are highly transforming but L801P at the SH3 domain is not. The acquired oncogenic activity of these mutants can be attributed to their enhanced activity as GEFs for Rho/Rac GTPases. Deciphering of the mechanisms leading to overactivity of the tested mutants revealed that the E59K mutation facilitates cleavage of a truncated protein that is uncontrollably active as a GEF, while D517E generates a highly stable overexpressed protein that is also more active as a GEF than wild-type Vav1. These findings support the classification of Vav1 as a bona fide oncogene in human cancer.

## Introduction

From the time that Vav1 was first identified as an oncogene (oncVav1), a question has remained as to whether it is mutated in human cancers, thus acting as a “real” oncogene. Vav was originally identified in an NIH3T3 screen for oncogenes^1^. The analysis of the Vav oncogene revealed that replacement of the amino terminus of wild-type Vav1 (herein called Vav1) by 67 residues of pSV2neo, a plasmid co-transfected as a selectable marker, led to its activation as an oncogene^1–3^. Wild-type Vav1, which is solely expressed in the hematopoietic system, functions as a cardinal signal transducer^4–9^. The best-known function of Vav1 is as a GDP/GTP exchange factor (GEF) for Rho/Rac GTPases, a function strictly controlled by tyrosine phosphorylation^10^. Rho/Rac activation causes cytoskeletal rearrangement that generates functional modifications in different cells, including immune cells^4–6,10^. Vav1 also participates in GEF-independent signaling pathways, including the c-Jun N-terminal kinase (JNK), extracellular signal–regulated kinase, nuclear factor-kappa B (NF-κB), and NFATc1 pathways, and associates with numerous adapter proteins, such as Shc, NCK, SLP-76, Grb2, and Crk^4^.

The physiological activity of Vav1 is fairly well understood, but its contribution to human cancers has only recently started to emerge. Many studies over the past decade have reported unexpected expression of the hematopoietic signal transducer Vav1 in a variety of human cancers. First, we reported the ectopic expression of Vav1 in human neuroblastoma tumors^11^. Subsequent studies from our laboratory and others reported aberrant expression of Vav1 in additional cancers, such as lung^12^, breast^13^, pancreatic^14^, and ovarian cancers^15,16^; esophageal squamous cell carcinoma^17^; and medulloblastoma^18^. Patients that carried pancreatic tumors expressing Vav1 had a poorer prognosis than those whose tumors were Vav1-negative^14,19^. Furthermore, Vav1 RNA interference was found to diminish proliferation of human pancreatic and lung cancer cell lines in vitro and in vivo, even in the presence of oncogenic K-Ras^12,14^. The identity of wild-type Vav1 from pancreatic cancer cell lines and tumors was confirmed by sequence analysis^14^. Thus the results obtained thus far undoubtedly indicate the importance of the abnormal expression of Vav1 in human cancers^20^, possibly via its activity as a GEF that regulates cytoskeletal organization or as a signal transducer that can affect production of growth factor or cytokines or both.

Artificially created molecular lesions, such as truncation of the amino terminus of Vav1 and mutation of tyrosine 174, were until recently the only known molecular lesions that converted Vav1 to a transforming gene in NIH3T3 fibroblasts^2,3^. The basis for this mechanism stems from the regulatory role of the amino terminus in the regulation of its GEF activity^21,22^. An additional activating mutation that was artificially introduced and is also found in human cancer was recently reported by Razanadrakoto et al.^23^. Thus this mutation at amino acid residue 797 (D797N) in the carboxy SH3 domain of Vav1 provides the protein with transforming properties^23^, albeit to a reduced extent than oncVav1. Numerous studies were conducted since the detection of Vav1 to untangle its structure/function by generating mutations at the Dbl homology domain (DH), pleckstrin domain (PH), C1, and both Src homology (SH) domains, SH3 and the SH2^24–27^. None of these experimentally introduced mutations yielded increased transformation of NIH3T3 fibroblasts, except the mutants mentioned above.

Current reports from human genome sequencing (Wellcome Trust Sanger Institute) indicate that Vav1 is mutated in ~1% of human cancers of numerous tissue origins (http://cancer.sanger.ac.uk). Mutations in Vav1 identified in human cancers span all of its cardinal domains. In addition, several recent publications report the identification of Vav1 mutations in human cancers, including various adult T cell leukemia/lymphoma^28–31^, as well as lung adenocarcinoma and squamous cell carcinomas^32^, but the activities of most of these mutants as transforming genes were not analyzed. It therefore remained to be tested whether or not the mutants in Vav1 that were identified in human cancers function as transforming genes under experimental conditions.

In this study, we investigated whether several amino acid substitutions in Vav1 at residues identified in human lung cancer patients acquire transforming properties, an attribute that can could credibly explain their potential involvement in human cancer.

## Results

### Vav1 mutants and their domain localization

**Fig. 1:**
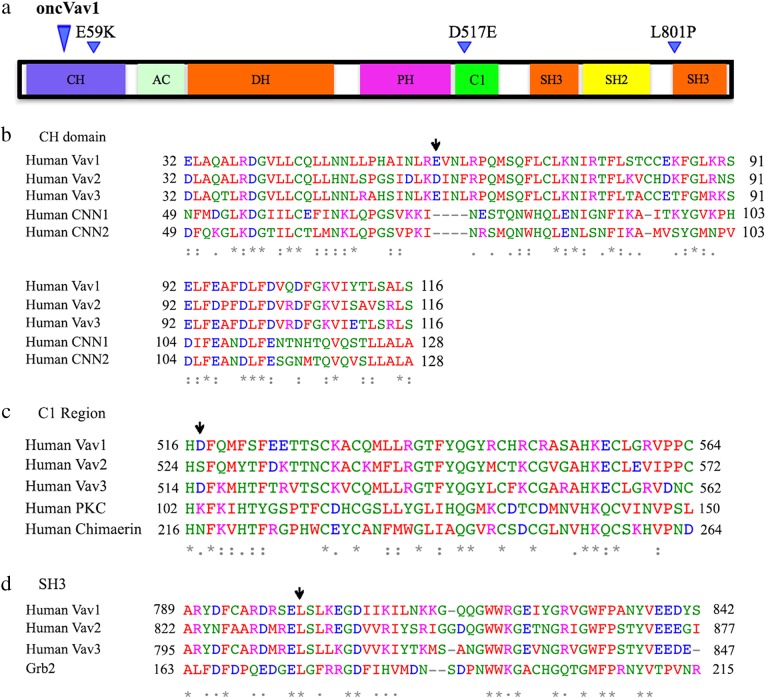
Vav1 structure and location of human cancer-identified mutations used in our study. **a** Vav1 encodes the following domains: a calponin-homology (CH) domain; an acidic (AC) motif, which contains three tyrosine residues; a DBL homology (DH) domain; a pleckstrin homology (PH) domain; a C1 domain; two SRC-homology-3 (SH3) domains; and one SRC-homology-2 (SH2) domain. The location of mutations introduced in Vav1 is indicated above the protein structure and the location of truncation of oncVav1. **b**–**d** Sequence comparisons of the various domains of Vav1 in which mutations were introduced: CH (**b**), C1 (**c**), and carboxy SH3 (**d**) to the same domains in other members of the human Vav1 proteins, as well as to additional proteins that encode the same domains. Below the protein sequences is a key denoting positions which have a single, fully conserved residue (*), (:) indicates conservation between groups of strongly similar properties, semi-conservative mutations, and (.) indicates conservation between groups of weakly similar properties. [Clustal FAQ #Symbols. Clustal. Retrieved 8 December 2014]. A black arrow points to the residue mutated in each domain represented in this figure. Each residue in the alignment is assigned a color if the amino acid profile of the alignment at that position meets some minimum criteria specific for the residue type as described (http://www.jalview.org/help/html/colourSchemes/clustal.html)

We chose to study the activity of Vav1 mutations from lung adenocarcinoma based on our previous studies^12,14^. Most of the confirmed mutations in lung adenocarcinoma patients were located at the calponin homology (CH) domain, the cysteine-rich C1 domain, the Src homology domain (SH2) and the carboxy SH3 domain. We introduced three mutations: E59K located at the CH domain, D517E at the C1 domain, and L801P at the carboxy SH3 domain (Fig. 1a).

The Vav1 CH domain is critically important for the involvement of Vav1 in calcium mobilization^33^. It also inhibits GEF activity, probably by binding the cysteine-rich C1 domain, suggesting that the inhibition occurs through an intramolecular interaction that occludes the DH domain and blocks access to its substrate GTPases^27^. The acidic glutamic residue (E) located at position 59 of the CH domain at the C1 domain is changed to a basic lysine residue (K) (Fig. 1b). Apparently, it does not show homology with the CH domain in other proteins, yet it is conserved in Vav3.

The D517E is located at the C1 domain. The Vav1 C1 domain is an atypical C1, which lacks the attributes required for lipid association and might instead be involved in protein–protein binding^34^. This domain plays a crucial function in the regulation of Vav1 activity through stabilization of the DH homology domain, probably through binding with the CH domain, thus obscuring the DH domain and preventing access to Rac/RhoGTPases. The amino acid residue aspartic acid (D) at position 517 was mutated to glutamic acid (E), both acidic residues. The mutation in the aspartic acid was introduced in an amino acid residue that is conserved in Vav3 but that shows only weak conservation with other residues located at the C1 domain of proteins, such as protein kinase C (Fig. 1c).

The third mutation was introduced in leucine 801, located at the carboxy-SH3 of Vav1 (Fig. 1d). The C-terminal SH3 domain of Vav1 binds to a varied number of proteins, such as RNA-binding proteins (hnRNP K, hnRNP C, and Sam68)^35,36^, cytoskeletal regulators (Zyxin)^37^, ubiquitination factors, viral proteins, various transcriptional modulators, and dynamin 2^4,5,38^. Leucine at position 801 was mutated to proline (P), both hydrophobic residues. This mutation was introduced in a position that is a fully conserved residue among the Vav proteins as well as in the SH3 of Grb2.

### Activity of Vav1 mutant proteins

**Fig. 2:**
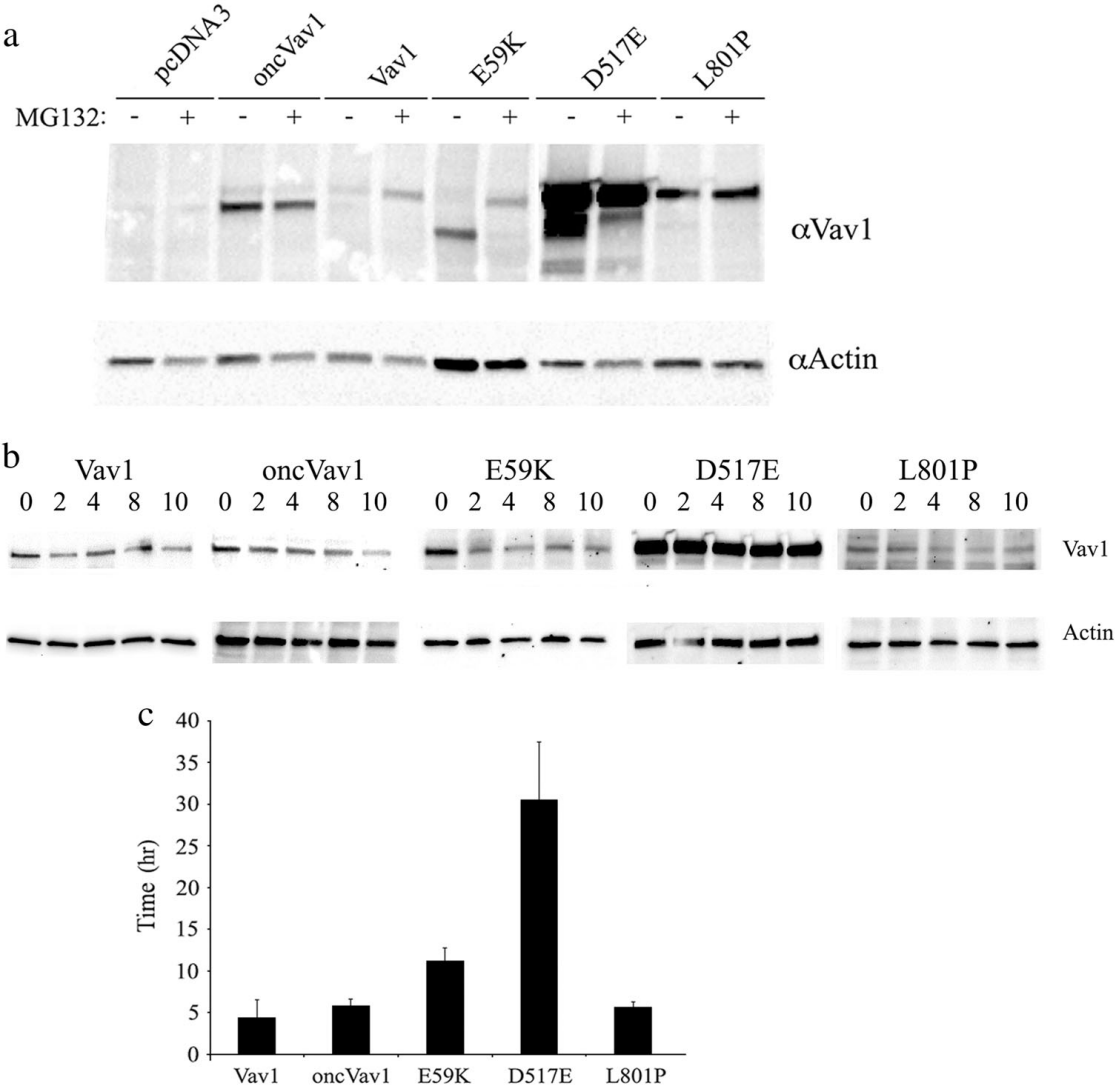
Protein properties of Vav1 mutants. **a** Expression of Vav1 in NIH3T3 cells stably transfected with pcDNA3, Vav1, and oncVav1 and the mutants E59K, D517E, and L801P was analyzed. Cells were treated with 25 μM of the proteasome inhibitor MG132 for 4 h or were left untreated. Cell lysates were immunoblotted with anti-Vav1 and anti-actin antibodies. **b** D517E is a highly stable protein. The half-life of Vav1 mutant proteins was measured by using CHX to block new protein synthesis. NIH3T3 cells stably expressing pcDNA3, Vav1, oncVav1, E59K, D517E, and L801P were treated with 100 μg/ml CHX for the indicated time periods. Cells were immunoblotted using anti-Vav1 and anti-actin antibodies. Results were quantified with the ImageJ program. **c** The histogram presents the mean time required for each protein to reach its half-life calculated by using http://www.calculator.net/half-life-calculator.html. Calculation was made for several time points. Error bars are indicated

The plasmids carrying our three generated mutants, E59K, D517E, and L801P, as well as plasmids encoding Vav1, oncVav1, and an empty vector (pcDNA3), were transfected into NIH3T3 fibroblasts and stable cell lines were generated. The mRNA levels of these Vav1 variants were similar (Supplementary Fig. 1). The cDNA of Vav1 in each of the transfected cells were sequenced to ensure that the appropriate mutants were expressed. Analysis of the proteins encoded by the various mutants revealed that, whereas mutants D517E and L801P produce proteins similar in size to the one generated by Vav1, mutant E59K yields a truncated protein smaller than the one produced by oncVav1, despite the fact that the length of the predicted protein sequence was the same as that of Vav1 (Fig. 2a). To examine the possibility that the truncated E59K protein is the result of the activity of enzymes controlled by the proteasome, we incubated the cells with MG132, a specific proteasome inhibitor (Fig. 2a). The size of the E59K protein produced under these conditions was similar to that of the protein produced by Vav1, thus suggesting that an enzyme regulated by the proteasome truncates it. All the other proteins remained unchanged. The identity of the cleaving enzyme is not yet known. An additional feature apparent from Fig. 2a is that D517E is overexpressed. We examined whether its pronounced expression is attributable to protein stability. To determine protein turnover, we incubated NIH3T3 cells expressing Vav1, oncVav1, and the mutants for various time points between 2 and 10 h (as indicated) with cycloheximide (CHX), which inhibits protein biosynthesis by preventing its translational elongation, and we then determined the protein expression levels in these cells (Fig. 2b). The calculated half-life of D517E is 30.55 h, seven times higher than that of Vav1 (4.4 h) (Fig. 2c). These results clearly indicated that, relative to the other Vav1 mutants and the Vav1 used in our study, the D517E mutation confers stability, thus providing a clue to its high expression.

**Fig. 3:**
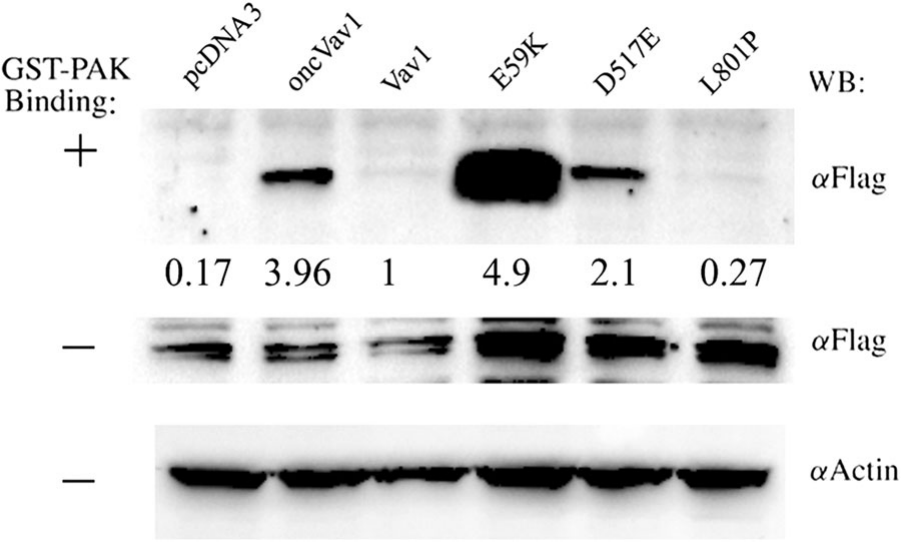
GEF activity of Vav1 mutant. The capacity of the Vav1 mutants E59K, D517E, and L801P to function as GEFs for Rac1 was analyzed. NIH3T3 cells stably expressing pcDNA3, Vav1, oncVav1, E59K, D517E, and L801P were transfected with FLAG epitope-tagged Rac1. Starved cell lysates were then incubated with the bacterial fusion protein that expresses Pak immobilized on glutathione-Sepharose beads. Bound proteins were separated on SDS−PAGE and immunoblotted (WB) with anti-FLAG mAbs, as indicated. Expression of transfected Vav1, Rac-FLAG, and actin was detected as indicated. The figure depicts one representative experiment of two experiments performed. Numbers outlined between the rows refer to the extent of Rac activation, calculated by the intensity of Rac-GTP bound to GST-Pak divided by the level of total Rac-Flag in each sample. The level of Rac activation by Vav1 was specified as 1

The importance of Vav1 in cancer is mainly due to its function as a GEF for Rho/Rac GTPases. Depletion of Vav1 in pancreatic cancer cells and in lung cancer demonstrated a reduction in Rac1 activity^12,14^. Furthermore, Fernandez-Zapico et al. showed that, unlike wild-type Vav1, a mutant of Vav1 that lacks its GEF activity cannot rescue the decline in proliferation of cancer cells of pancreatic cells depleted of Vav1^14^. The activation of Vav1 as a GEF in T cells is strictly controlled by tyrosine phosphorylation^39^. Furthermore, truncated Vav1 proteins have been shown to be constitutively active toward Rac1^39^. Our analysis of the ability of the various mutant proteins to activate Rac1 indeed revealed that oncVav1, E59K, and D517E activate Rac1-GTP to various degrees and that the truncated protein E59K is even more active than oncVav1 (Fig. 3). Needless to say, E59K, D517E, and oncVav1 were more active than Vav1. It is worth noting that our experiments were performed with starved cells without the addition of growth factors and thus revealed the ability of the mutant proteins to activate Rac1 even without stimulation (Fig. 3b).

**Fig. 4:**
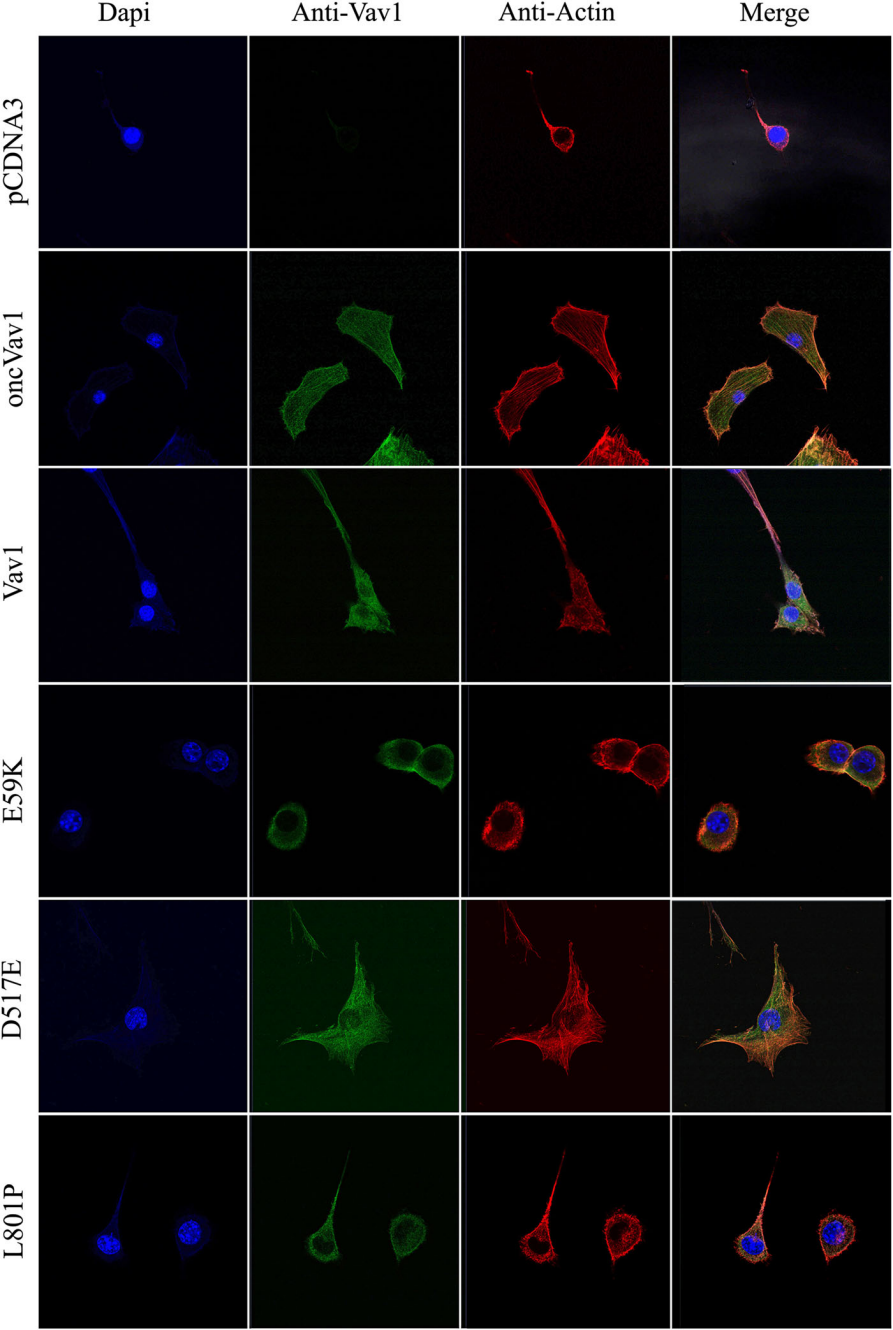
Morphology of NIH3T3 cells stably expressing pcDNA3, Vav1, oncVav1, E59K, D517E, and L801P. Vav1 protein (green) was detected by anti-Vav1 mAbs, actin filaments were detected by phalloidin (red), and nuclei were stained with DAPI (blue). Representative photographs were taken with a Zeiss LSM 710 confocal microscope

We then examined whether the various Vav1 mutants induce changes in cell morphology, as inferred from their activity as GEFs toward Rac1. NIH3T3 cells expressing oncVav1 exhibited morphology reminiscent of transformed cells resulting in many large cells with numerous cytoplasmic extensions, whereas NIH3T3 cells expressing either pcDNA3 or Vav1 showed no remarkable morphological changes (Fig. 4). Compared to control cells, NIH3T3 cells expressing either E59K or D517E also exhibited altered morphologies (Fig. 4), reminiscent of transformed NIH3T3 cells such as those expressing oncVav1. Notably, NIH3T3 cells expressing the Vav1 mutant L801P displayed similar morphology to control cells and those expressing Vav1 (Fig. 4). These results clearly demonstrated that the presence of mutants E59K and D517E leads to changed cell morphologies that are indicative of transformed cells, probably owing to their increased GEF activity.

#### Transforming potential of cancer-identified mutant Vav1 proteins

We examined additional Vav1-related biological characteristics, beginning by analyzing cell proliferation using the MTT ((3-(4,5-dimethylthiazol-2-yl)-2,5-diphenyltetrazolium bromide) assay, which assesses cell metabolic activity indicative of cell proliferation (Supplementary Fig. 2). Our results clearly indicated that our generated cells expressing oncVav1, E59K, and D517E proliferate at significantly higher rates than all of the other generated cell lines expressing pcDNA3, Vav1, and L801P (Supplementary Fig. 2). Among the cells showing high proliferation, a hierarchy of proliferation rates could be drawn, with E59K > D517E > oncVav1 (Supplementary Fig. 2). Notably, the growth-proliferation rates of cells expressing the mutant Vav1 L801P were similar to those of cells expressing Vav1 (Supplementary Fig. 2). These results clearly demonstrated that both of the Vav1 mutants E59K and D517E endow the cells with very high proliferation rates, suggesting that these cells acquire properties that differ from those of cells expressing Vav1.

**Fig. 5:**
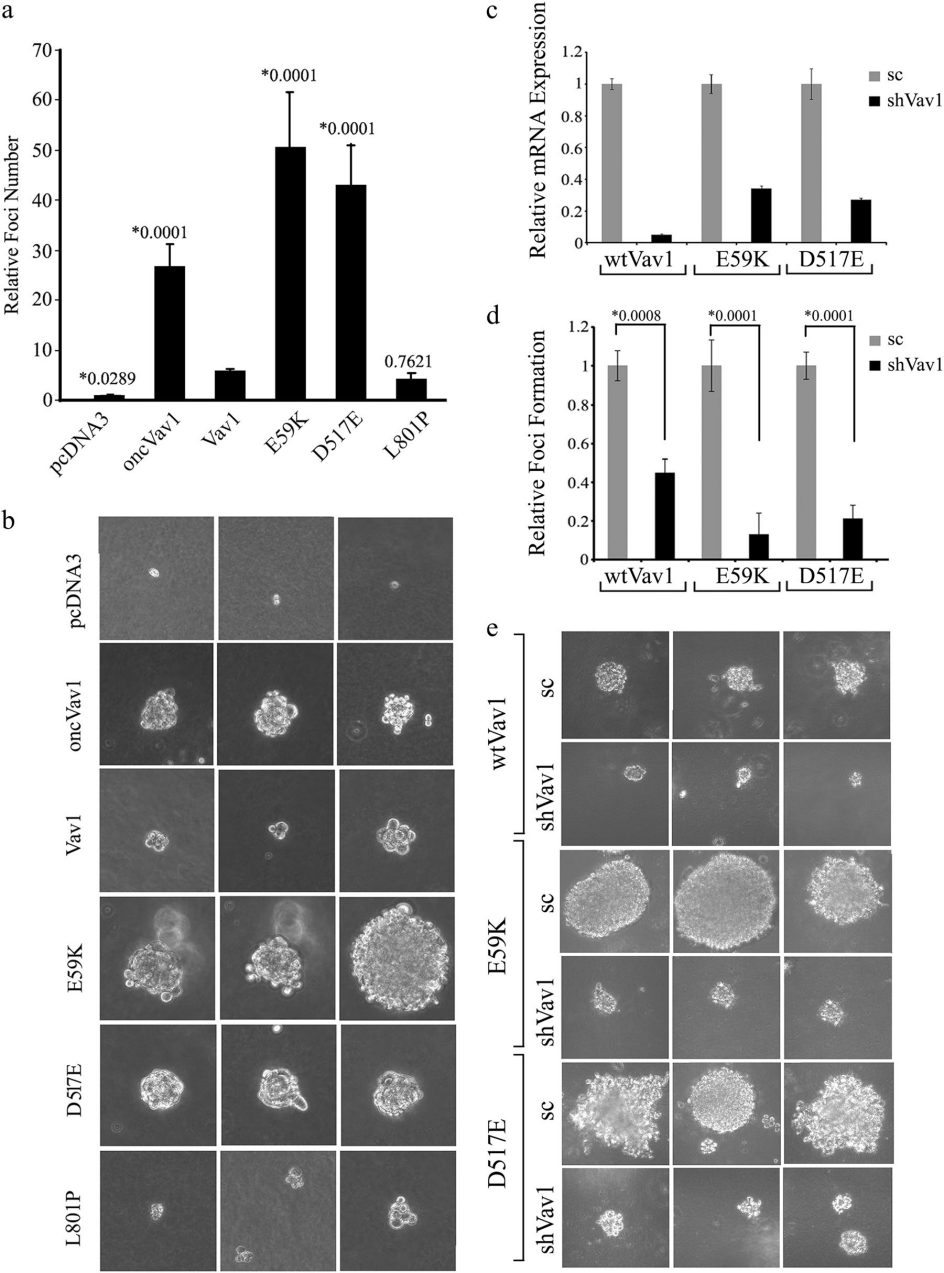
Transforming activity of Vav1 mutants in vitro. **a** NIH3T3 cells stably expressing pcDNA3, Vav1, oncVav1, E59K, D517E, and L801P were suspended in DMEM medium containing 0.3% agar and 10% calf serum and plated onto a bottom layer with 0.8% agar. 1 × 10^5^ cells were plated in a well in a 6-well plate in triplicate and the number of foci were counted 14 days later. The histogram presents means ± S.E. of triplicate values from three independent experiments. Unpaired Student’s *t* test (*) compare the number of foci obtained in each cell line to cells expressing Vav1. **b** Representative photographs of three foci from each transfected cell line in **a** are presented. **c** Vav1 was silenced in NIH3T3 cells stably expressing wtVav1, E59K, and D517E using shRNA sequences against wtVav1 (shVav1) or shscrambled (sc). The silencing efficiency of Vav1 was measured by real-time PCR using specific primers (supplementary Table 1). UBC and HPRT were used as a control. **d** NIH3T3 cells stably expressing Vav1, E59K, and D517E and cells in which Vav1 was silenced were analyzed for foci formation as detailed in **a**. The histogram presents means ± S.E. of triplicate values from two independent experiments. Unpaired Student’s *t* test was performed as indicated above. **e** Representative photographs of three foci from each cell line in **d** are presented

A focus-forming assay in soft agar corroborated the MTT findings (Fig. 5a, b). When grown on soft agar, NIH3T3 cells expressing either the E59K or the D517E Vav1 mutant formed significantly more foci than cells expressing pcDNA3, Vav1, or L801P (Fig. 5a), thus exhibiting a transforming potential exceeding that of Vav1 and even that of oncVav1. The foci produced by these mutants, as well as those generated by oncVav1, were larger than those generated by cells expressing pcDNA3, Vav1, or L801P (Fig. 5b). Single subclones generated by limiting dilution exhibited comparable transforming potential (supplementary Fig. 3). To further substantiate our results, we silenced the expression of Vav1 in cells expressing wtVav1 (95%), E59K (66%), and D517E (74%) by using a virus containing Vav1 short hairpin RNA (shRNA) sequences (Fig. 5c). The reduction in wtVav1 or the mutants E59K and D517 led to a marked decrease in foci formation and foci size (Fig. 5d, e). These results further emphasized the marked transforming potential of the Vav1 mutants E59K and D517E, a feature not evidenced up to now by other cancer-detected Vav1 mutants^40^.

#### Generation of tumors by the cancer-identified mutant Vav1 proteins in a xenograft model

**Fig. 6:**
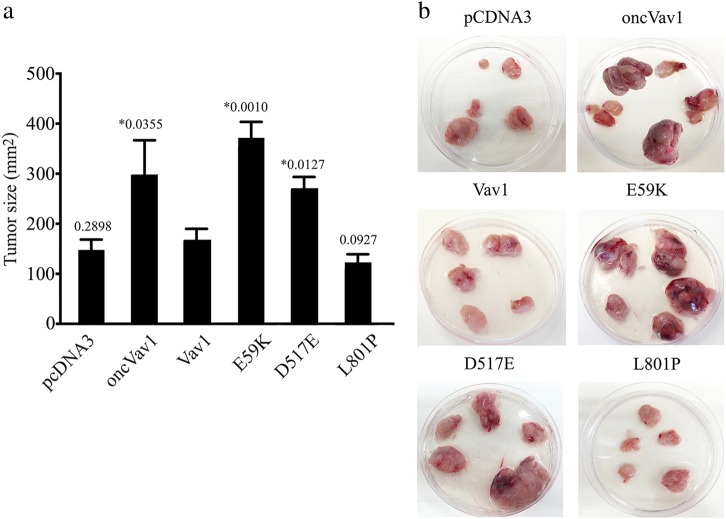
Transforming activity of Vav1 mutants in vivo. **a** NIH3T3 cells stably expressing pcDNA3, Vav1, oncVav1, E59K, D517E, and L801P were injected subcutaneously into athymic female NOD/SCID mice. The histogram shows the mean tumor sizes of 3 experiments 30 days following injection ( ± SE). Six mice were used for each group in each experiment. Differences between tumors generated by oncVav1, E59K, D517E, and Vav1 are, as indicated, highly significant (*unpaired Student’s *t* test). **b** Photographs of representative tumors 30 days post-injection are depicted

To examine the tumorigenic effect of Vav1 mutant expression in a xenograft in vivo model, we injected NIH3T3 cells transfected with the Vav1 mutants E59K, D517E, and L801P, as well as those transfected with pcDNA3, oncVav1, and Vav1, subcutaneously into the flanks of athymic non-obese diabetic/severe combined immunodeficient (NOD/SCID) mice and followed the appearance and growth rates of the tumors generated (Fig. 6a, *n* = 6 in each group). Cells expressing the E59K and D517E Vav1 mutants resulted in markedly increased tumor sizes compared to tumors generated by NIH3T3 cells expressing pcDNA3, Vav1, or L801P (Fig. 6a, b). These results convincingly attested to the high transforming potential of the Vav1 mutants E59K and D517E.

**Fig. 7:**
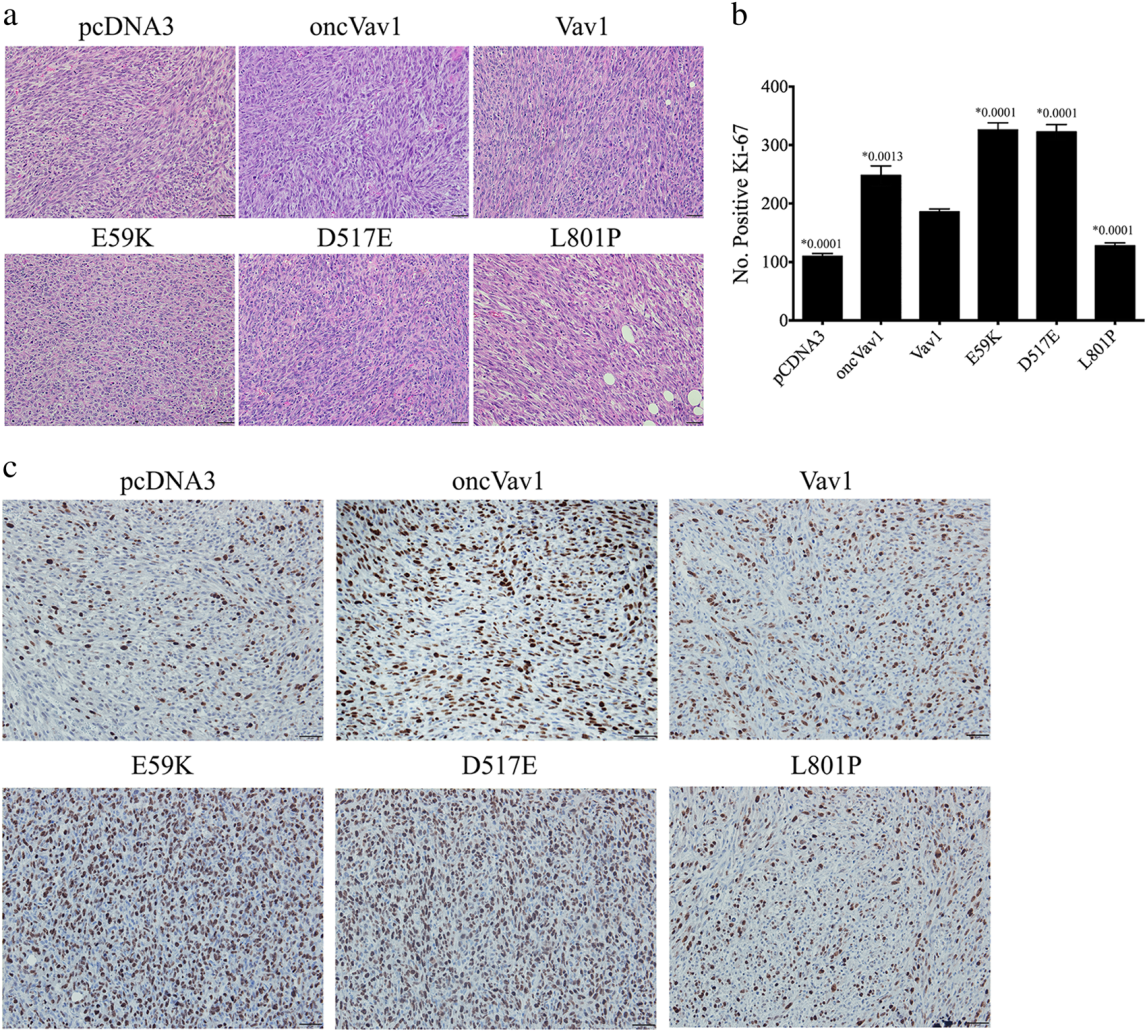
Analysis of nude mouse tumors generated by NIH3T3 cell expression. **a** Representative pictures of H&E-stained tumors generated by NIH3T3 cells stably expressing pcDNA3, Vav1, oncVav1, E59K, D517E, and L801P in NOD/SCID mice. **b** Histogram showing the calculated numbers of Ki-67-positive cells in tumors of NIH3T3 cells stably expressing pcDNA3, Vav1, oncVav1, E59K, D517E, and L801P. The *p* value for each of these groups compared to the Vav1 group is recorded above each column. **c** Representative pictures of tumors generated by NIH3T3 cells expressing pcDNA3, oncVav1, E59K, D517E, and L801P stained with anti-Ki-67 antibodies

Resected tumors were submitted for histological analysis of hematoxylin and eosin (H&E)-stained slides (Fig. 7a) and for cell proliferation (Fig. 7b, c). Most cell lines generated a classic fibrosarcoma herringbone pattern. E59K-transformed cells, however, formed anaplastic high-grade sarcomas with epithelioid features. Immunostaining for the proliferation marker Ki-67 showed that tumors generated by the NIH3T3 cells transfected with E59K, D517E, or oncVav1 exhibited significantly higher indices of proliferation than tumors expressing pcDNA3, Vav1, or L801P (Fig. 7b, c). These results further substantiated our findings that the Vav1 mutants E59K and D517E convert the proto-oncogene to highly transforming proteins that are even more active than oncogenic Vav1, whereas the L801P mutant is non-transforming.

**Fig. 8:**
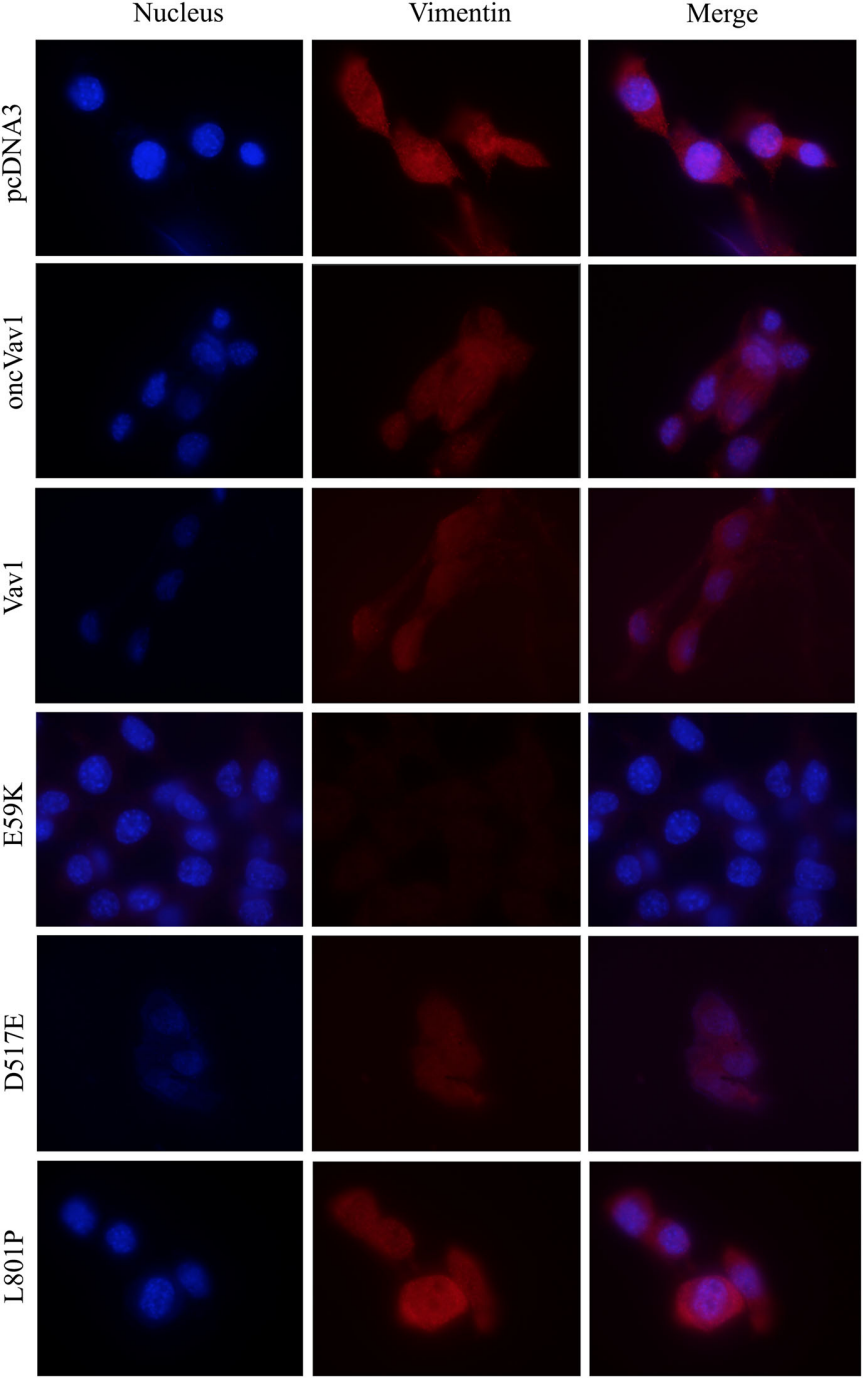
Changes in expression of the mesenchymal marker vimentin in cells expressing Vav1-mutant proteins. Vimentin expression (red) was detected by immunofluorescence in NIH3T3 cells stably expressing pcDNA3, Vav1, oncVav1, E59K, D517E, and L801P. Nuclei are stained with DAPI (blue). Representative photographs indicating the differences in morphology of the cells were taken with Nikon eclipse 90i confocal microscope and analyzed by the NES element program

**Fig. 9:**
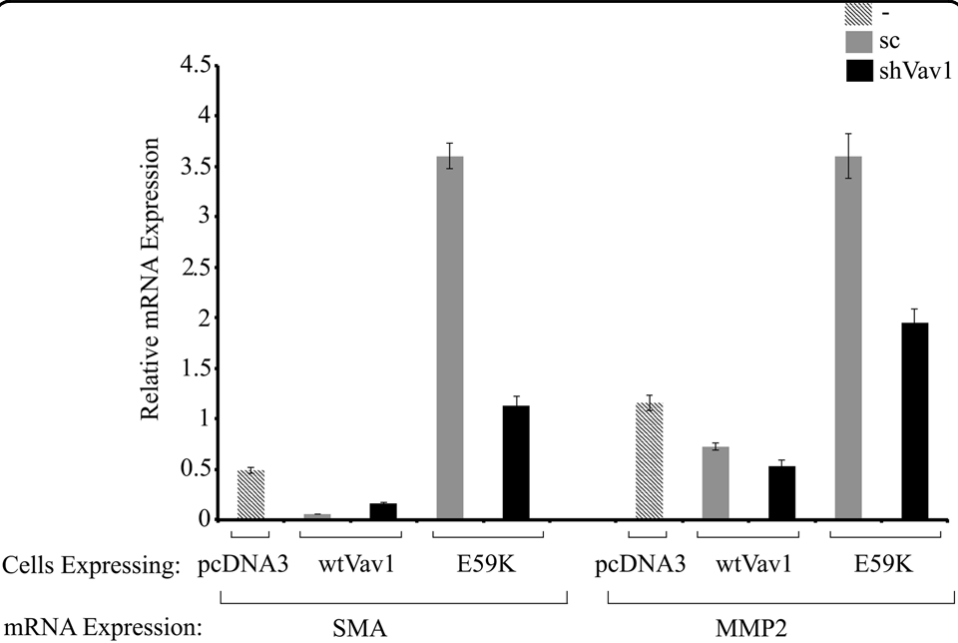
Changes in expression of the different markers in cells expressing Vav1-mutant proteins. NIH3T3 cells stably expressing pcDNA3, wtVav1, and E59K with depletion of VAV1 expression (shVav1) or without (scrambled, sc) were subjected to real-time PCR. SMA and MMP2 expression was measured using specific primers (Supplementary Table 1). UBC and HPRT were used as a control

To determine the nature of the transformed NIH3T3 cell lines generated in this study, we analyzed the expression of the mesenchymal marker vimentin (Fig. 8). Vimentin is a 57-kD, type III intermediate filament that is found in the mesenchymal cells of various types of tissues during their developmental stages and that maintains cell and tissue integrity^41^. Because of these properties, vimentin is often used as a marker of mesenchymally derived cells or of cells undergoing epithelial-to-mesenchymal transition during both normal development and metastatic progression^42^. From representative pictures of the vimentin-stained cells used in our study, it was apparent that the level of vimentin expression is reversibly correlated with the cells’ transformation state. Thus, whereas NIH3T3 fibroblasts transfected with pcDNA3 exhibited a high level of vimentin corresponding to their mesenchymal origin, cells expressing the E59K protein lost their vimentin expression (Fig. 8). Furthermore, NIH3T3 fibroblasts transfected with Vav1 or oncVav1 expressed vimentin but to a lower extent than those transfected with pcDNA3 or the Vav1 mutant L801P. We analyzed whether additional cancer aggressiveness markers change in cell expressing E59K (Fig. 9). Alpha smooth muscle actin (α-SMA), a member of actin proteins, which is increased in cancers such as breast cancer^43,44^, and matrix metalloproteinase2 (MMP2) that serves as a biomarker for epithelial–mesenchymal transitions^45^ were significantly increased in E59K-expressing cells compared to Vav1- or pcDNA3-expressing cells. Remarkably, the increased expression of α-SMA and MMP2 was considerably reduced upon Vav1 silencing (Fig. 5c), albeit baseline expression was not restored completely (Fig. 9). A similar pattern was observed also in cells expressing Vav1. Our results clearly point to changes in tissue differentiation markers indicative of transforming phenotypes.

## Discussion

Our study strongly supports the classification of Vav1 as a bona fide oncogene in human cancer. We showed that mutants E59K and D517E, both of which have been detected in human lung cancer, are highly transforming in the experimental system used here (Figs. 5−9). We chose to analyze the activity of Vav1 mutants by means of an NIH3T3 transformation assay that was used some 30 years ago to identify oncogenes, such as Ras^45,46^. The ability of Ras and other oncogenes to transform NIH3T3 cells relies on the immortal state of these cells^47^. On the other hand, at least two oncogenes are necessary for transformation of mouse embryonic fibroblasts (MEFs)^48^. NIH3T3 cells originating from MEFs were shown to spontaneously become immortalized and overcome cellular senescence, when under certain culture conditions^49^. Newbold and Overell showed that acquiring the immortal state by these cells was a necessary but not a sufficient requirement for malignant transformation, in line with the multistage model of cancer^50^. Based on these accumulating insights, it is highly likely that Vav1 mutants such as E59K and D517E need the cooperation of other transforming genes to achieve malignant transformation in human tumors. The ability of the Vav1 mutants E59K and D517E to fully transform NIH3T3 cells suffices to classify them as oncogenic proteins.

Evidently, the mechanisms conferring oncogenic potential on the two point mutations E59K and D517E are different. E59K produces a truncated protein, whereas D517E manifests dramatic overexpression. Interestingly, proteolysis of Vav1 has been reported in several studies: it was shown to undergo Cbl-dependent ubiquitination in T cells^51,52^ and it was identified as a caspase substrate during apoptosis in lymphoid cells^53^. In vivo, Vav1 is cleaved in T cells at the conserved caspase consensus cleavage site DLYD161C, leading to a carboxy-terminal cleavage product Vav1p76, which exhibits an intermediate stability^53^. Vav1 cleavage in T cells is prevented by the caspase inhibitor zVAD^53^. In NIH3T3 cells, however, E59K generates a stable protein that is refractory to zVAD inhibition (Supplementary Fig. 4), thus negating its cleavage by caspase. It is thus clear that the E59K mutation facilitates cleavage by an unidentified enzyme generating a highly active protein. Based on our results, we suggest that the process is regulated by proteasome machinery, which usually, as is well known, fully degrades its substrates. Nevertheless, there are several reported cases in which the proteasome is responsible for only partial truncation of proteins. For instance, the p50 subunit of NF-κB is generated from processing of the p105 precursor by ubiquitin- and proteasome-mediated processes^46,55^. The ubiquitin ligase KPC1 was recently shown to mediate this partial truncation of NF-κB^54^. Other examples include eIF4G and eIF3a, which are endoproteolytically cleaved by the 20S proteasome^55^. Inhibition of the proteolytic activity of the 20S proteasome by specific inhibitors prevents cleavage of both factors^57^. Also, Constantinou and colleagues demonstrated that the translational inhibitor 4E‐BP1 is cleaved following p53 activation by a mechanism that is dependent on the proteasome^56^. In addition, it was demonstrated that a proteolytic cleavage by the 20S proteasome leads to the generation of a split-off C-terminal 105-amino acid-long YB-1 fragment^57^.

It is interesting to note that the GEF activity of E59K is highly elevated compared to Vav1 and even compared to oncVav1 (Fig. 3b); notably too, the activity of Vav1 as a GEF is cardinal for its transforming potential^12,14^. The importance of this activity for Vav1 function was previously demonstrated in pancreatic and lung cancers, where it is ectopically overexpressed^12,14^. The raised GEF activity of Vav1 most probably stems from its truncation. Given the elevated activity of E59K as a GEF, it is conceivable that this protein loses a portion of its amino terminus, a well-documented event that leads to induction of an oncogenic form of Vav1 owing to loss of the regulatory mechanism and generation of a protein that functions uncontrollably as a GEF^40^. Accordingly, the activity of the E59K mutant also leads to the appearance of aggressive tumors in NOD/SCID mice, further highlighting the aggressive nature of this protein’s transforming potential. In contrast, the D517E mutation results in Vav1 stabilization, leading to remarkably high levels of the full-length protein (Fig. 2). These results are in agreement with numerous reports suggesting that the oncogenic activity of Vav1 could stem from its overexpression, as demonstrated in vitro in NIH3T3 fibroblasts, or when it is ectopically expressed in vivo in human cancer^2,3,12,14^. The L801P mutation, albeit detected in human tumors, did not show any oncogenic activity in our study. It is possible that in a different cellular system, or in an appropriate microenvironment, it might be oncogenic in conjunction with additional transforming events. However, given the strong transforming potential of other Vav1 mutations in this study, it is also possible that L801P is a passenger mutation. Our results thus categorically demonstrate the need for analysis of each of the Vav1 mutations detected in human cancers, since not all Vav1 mutants have transforming properties, as demonstrated here with respect to L801P.

The transforming activity of Vav1 mutants detected in human cancers has been tested in only a limited number of studies. For instance, Vav1-GSS, a rearranged Vav1 gene that was identified in a screen of peripheral T cell lymphomas (PTCLs) and NOS (not otherwise specified) and anaplastic large cell lymphomas, was shown to promote cell growth and migration in a Rac1-dependent manner^30^. Also, spliced variants of Vav1 (VAV1 Δ778–786), as well as novel Vav1 gene fusions (*VAV1-THAP4*, *VAV1-MYO1F*, and *VAV1-S100A7*) found in PTCLs, demonstrate elevated activity of Vav1-dependent and non-dependent GEF functions^29^. Vallois et al. detected frameshift deletions or missense mutations in four patients, three of them with angioimmunoblastic T cell lymphoma and one with thyroid follicular hypertrophy-like PTCL^31^. These mutations, which generate changes in the acidic domain, the SH2 domain, the C1 domain. and the SH3–SH2–SH3 module, have been shown to enhance T cell activity^58^. Importantly, however, the ability of these Vav1 variants to transform cells was not examined in any of those studies. As previously demonstrated, the activity of Vav1 mutants as oncogenic cannot be presumed on the basis of their activities in T cells^59^. Other notable mutations in Vav1 were shown to cluster at several hotspot amino acids in the acidic (Tyr 174 and Glu175), PH (Lys404), C1 (Glu556), and SH3 (Arg798 and Arg822) domains, 18% of them identified in T cell leukemia/lymphoma, but again both the functional activity of these mutants and their potential oncogenic activity remain to be tested^28^. Finally, exome sequencing of 660 lung adenocarcinomas revealed mutations in Vav1^32^. In that study, the mutations identified in the protein were not examined for changes in their biochemical or biological activity, yet it was predicted that these are activating mutations. For example, a mutation that converts serine 67 to tyrosine near the interface of the CH, AC, and PH domains was presumed to augment the protein’s activity, as it had previously been shown to increase overall GEF activity^60^.

Robles-Valero et al. reported that loss of Vav1 in immature T cells facilitates the activity of Notch1 fragment (ICN1), leading to the development of T cell acute lymphoblastic leukemia^61^. No reports confirm this classification of Vav1 as a potential tumor suppressor in T cells nor has it yet been shown in other cancers, especially in solid tumors of epithelial origin. However, the idea that the same tumor gene can be classified as an oncogene or a tumor-suppressor gene, depending on the physiologic context, has been previously raised. As a well-known example of such dual activity, the tumor-suppressor gene p53 can also function as an oncogene when it carries a gain-of-function mutation that contributes to the tumorigenic properties of cancer cells^62^. We have previously shown that Vav1 plays a pro-apoptotic role in breast cancer cells that express p53, while it functions as an antiapoptotic protein when breast cancer cells lack the expression of p53^13^. Also, several studies indicate that Notch1 functions as an oncogene in hematological malignancies such as lymphomas and leukemias^63^, whereas it is suggested to operate as a tumor-suppressor gene in certain solid tumors^64^. A similar phenomenon was recently suggested when delineating the molecular pathogenesis of hepatocellular carcinoma (HCC), where experiments in mouse tumor models revealed that removal of pro-tumorigenic genes such as MET, NF-κB, Stat3, Jnk, Shp2, and β-catenin paradoxically enhance HCC development under certain conditions. Thus opposing roles in promoting and suppressing HCC was demonstrated for the same molecules in different animal models^65^.

## Materials and methods

### Cloning and vectors

Vav1, oncVav1 generated by a 45 amino acid deletion at amino terminus, and the mutants E59K, D517E, and L801P obtained from Novartis were sub-cloned into a pcDNA3.0 vector that carries the neomycin gene as a selectable marker.

### Cell culture and transfection

NIH3T3 cells (mycoplasma free) were grown in Dulbecco’s modified Eagle’s medium containing 10% fetal calf serum (Biological Industries, Israel). The cells were stably transfected with the following plasmids (6 μg): pcDNA3 as control; Vav1, oncVav1, E59K, D517E, and L801P using the jetPEI^®^ transfection reagent (PolyPlus, CA) according to the manufacturer’s instructions. Stable clones were selected using 0.5 mg/ml G418 and were verified by sequencing. We further applied limiting dilution to all stable clones by seeding one cell per well in 96-well plates and selected two subclones for each mutation. For caspase inhibition, the cells were treated with 50 μM zVAD, a caspase inhibitor overnight.

### Silencing gene expression by shRNA

Cells were infected with pLKO-based (Open Biosystems) lentiviral vector that contains the human Vav1- shRNA (shVav1) or scrambled (sc) encoding sequences (Table S1). Infected cells were selected with puromycin.

### Quantitative real-time PCR

Total RNA and cDNAs were prepared as detailed for reverse transcriptase-PCR. Detection of VAV1, SMA, and MMP2 was performed using cyber green PCR master mix (BioRad, CA, USA) and the required primers (Table S1). Real-time PCR was performed by using the ABI Prism 7300 real-time PCR technology (Applied Biosystems, CA, USA). Three independent experiments were performed, and in each experiment triplicates were used.

### RNA extraction and PCR analysis

Total RNA was purified from NIH3T3 cells stably expressing pcDNA3, Vav1, oncVav1, E59K, D517E, and L801P using a Tri reagents procedure (SIGMA) according to the manufacturer’s instruction. cDNA was made using qScript cDNA Synthesis Kit (Quanta Biosciences) and was subjected to PCR analysis.

### Proteasome inhibition

Proteasome inhibition was induced by the addition of 25 μM MG132 (carbobenzoxy-Leu-Leu-leucinal) inhibitor (AGC Scientific, CA). After incubation for 4 h, the cells were lysed and subjected to immunoblotting.

### Immunoblotting assay

Cell lysis and immunoblotting procedures were performed as described previously^36^. The antibodies used were monoclonal anti-Vav1 (for western blotting, immunohistochemistry, and immunofluorescence; #05-219, Upstate Biotechnology, NY), anti-Flag (#087K6002, Sigma, Israel), and anti-actin (#8224, Abcam, USA).

### Immunofluorescence assay

Immunofluorescence was performed as previously detailed^12^ using anti-Vav1 monoclonal antibodies (mAbs) and secondary AlexaFluor-647 anti-mouse IgG, anti-Alexa fluor-546 phalloidin staining for actin cytoskeleton (Molecular Probes, USA; #A22283), anti-vimentin (Progen GP53; Germany), and Cy3 donkey anti-guinea pig (Jackson Laboratories, ME#705-165-148). Nuclear staining was detected by Dapi.

### Immunohistochemistry

Mice were euthanized and their tumors were dissected, fixed in formalin, and embedded in paraffin. Formalin-fixed and paraffin-embedded sections were immunostained with the primary antibody against Ki-67 (ThermoScientific, MA), according to the manufacturer’s instructions. Antigens were retrieved in 25 mM citrate buffer, pH 6.0, by heating of samples to 125 °C for 3 min in a decloaking chamber (Biocare Medical, CA).

### Analysis of GEF activity of Vav1

Cells generated in our study (6 × 10^5^) were transfected with 4 μg of FLAG epitope-tagged Rac plasmid. Rac activity was tested by using the Rac Activation Kit (ab139586, Abcam, USA).

### MTT cell proliferation assay

Cells generated in our study were cultured in 6-well plates, grown to sub-confluence, and then starved for 48 h. During the starvation period, 0.1 mg/ml of MTT in dimethyl sulfoxide was added to triplicate wells of each cell line and analyzed at various time intervals. Absorbance was quantified at 540 nm.

### Assay of soft agar colony formation

Soft agar colonies were assayed in six-well culture dishes. The bottom of each well was coated with 3 ml of medium containing 0.8% soft agar. Triplicates, each containing 1 × 10^5^ cells, were plated on top in 3 ml of medium containing 0.3% soft agar. Experiments were reproduced three times. Cells were fed every third day with 0.3 ml of medium, and colonies were counted after 14 days^12^.

### Tumorigenicity assay using NOD/SCID mice

NIH3T3 cells (*n* = 2 × 10^6^) expressing pcDNA3 as control, Vav1, oncVav1, and the different Vav1 mutants were injected subcutaneously, together with Matrigel (BD Biosciences, CA), into 10-week-old NOD/SCID mice. Mice were randomly allocated to each group without any bias to age, sex, or any other criteria. The sample size was calculated by G power 3.1 calculator. No blinding was used. Tumor growth was measured twice weekly. Tumor size was calculated as the product of width and length (mm^2^). Mice were euthanized 20 days post-injection and the excised tumors were each subjected to H&E staining and immunohistochemistry using anti-Ki67 antibodies. Experiments were performed three times with five mice in each group. Staining of specimens was evaluated by a board-certified pathologist (E.P.). These experiments were carried out according to NIH guidelines and all procedures were approved by the Animal Facility at the Hebrew University.

### CHX assay

Cells (*n* = 6 × 10^5^) were seeded in 6-cm plates. The next day, the cells were treated with 100 μg/ml of CHX (Sigma C7698) for the indicated times. At each time point, cells were lysed and subjected to immunoblotting for Vav1 and actin. Immunoblots were quantified using ImageJ analysis. At each time point, the value of Vav1 protein was normalized by that of actin. Time point 0 was considered as 1. The histogram presents the time required for each protein to reach its half-life calculated by using http://www.calculator.net/half-life-calculator.html. Calculation was made for several time points.

## Electronic supplementary material

Supplementary data
