# Supplementary material for: Vav1 mutations identified in human cancers give rise to different oncogenic phenotypes
Source: Oncogenesis. 2018 Oct 8;7(10):80. doi: 10.1038/s41389-018-0091-1 (PMC6175932; doi:10.1038/s41389-018-0091-1)
Supplement: Supplementary file 1 — Supplementary data [file 41389_2018_91_MOESM1_ESM.pdf]

Supplementary Materials

Supplementary Fig. 1

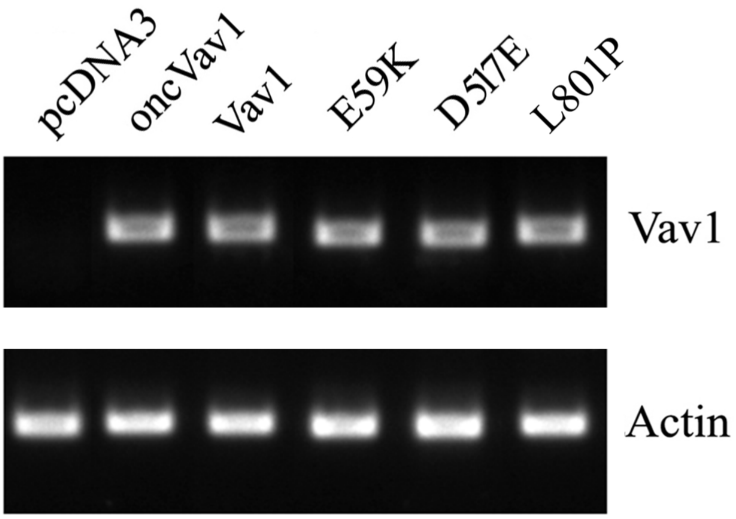

Supplementary Fig 1. *The level of Vav1 mRNA expression is comparable among NIH3T3 cells stably expressing Vav1, oncVav1, E59K, D517E and L801P.* Vav1 mRNA levels in NIH3T3 cells expressing the various mutants used in our study was subjected to PCR analysis using primers for Vav1 and actin as a loading control.

Supplementary Fig. 2

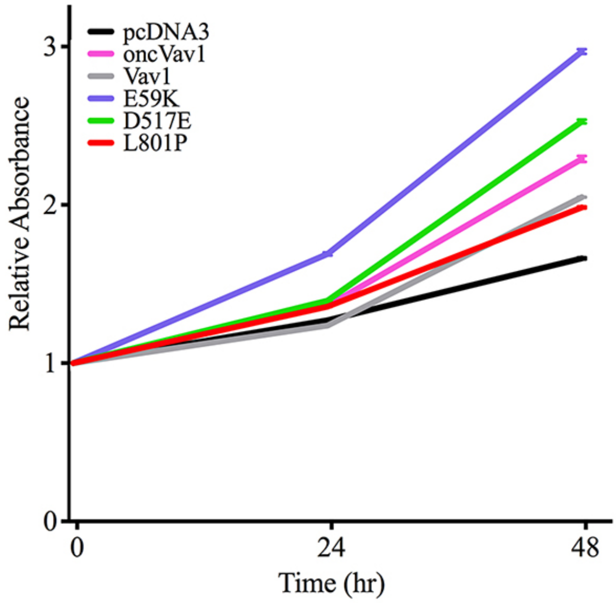

Supplementary Fig 2. *MTT assay of NIH3T3 cells stably expressing pcDNA3, Vav1, oncVav1, E59K, D517E and L801P.* Data show mean absorbance at 550 nm (relative to 24-hour value) of 3 independent experiments. Error bars are indicated. Statistical analysis indicates $p<0.05$ values between E59K, D517E and oncVav1 to Vav1 expressing cells.

Supplementary Fig. 3

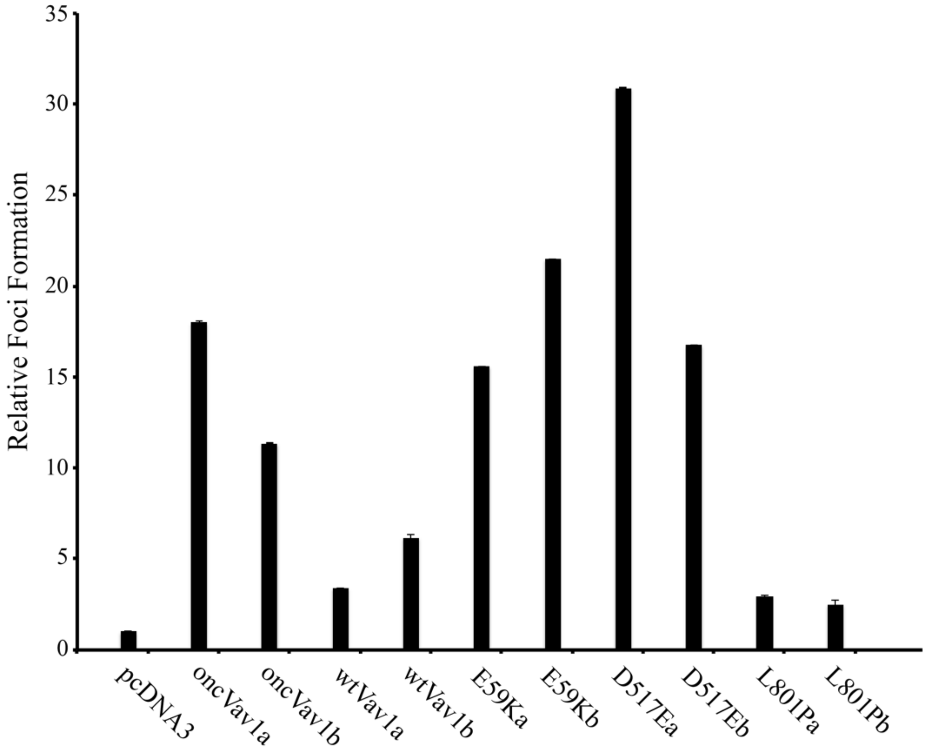

Supplementary Fig 3. *Expression of Vav1 mutant protein in the presence of caspase and proteasome inhibitors.* NIH3T3 cells stably transfected with pcDNA3, Vav1, oncVav1, and the mutants E59K, D517E and L801P were treated overnight with the caspase inhibitor zVAD (50 µM). The next day the same cells were further incubated with 25 µM of the proteasome inhibitor MG132 for 4 h or left untreated. Cell lysates were subjected to immunoblotting with anti-Vav1 and anti-actin antibodies.

Supplementary Fig. 4

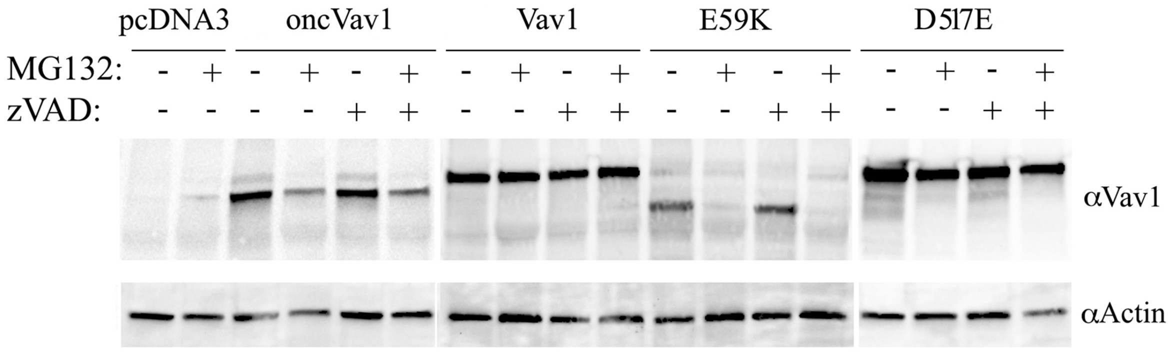

Supplementary Fig 4. *Expression of Vav1 mutant protein in the presence of caspase and proteasome inhibitors.* NIH3T3 cells stably transfected with pcDNA3, Vav1, oncVav1, and the mutants E59K, D517E and L801P were treated overnight with the caspase inhibitor zVAD (50 μM). The next day the same cells were further incubated with 25 μM of the proteasome inhibitor MG132 for 4 h or left untreated. Cell lysates were subjected to immunoblotting with anti-Vav1 and anti-actin antibodies.

Supplementary Table 1: Primer sequences used in our study

**Realtime**		
Gene	Forward primer	Reverse primer
SMA	5'-TGACTACTGCCGAGCGTGA-3'	5'-GTGATCACCTGCCCGTCA-3'
MMP2	5'-TTTGCTCGGGCCTTAAAAGTAT-3'	5'-CCATCAAACGGGTATCCATCTC-3'
hVAV1	5'-GCATTAAATATAACGTCGAG-3'	5'-CTTTAGAGAGTTCTGCTGGT-3'
UBC	5'-CAGCCGTATATCTTCCCAGACT-3'	5'-CTCAGAGGGATGCCAGTAATCTA-3'
HPRT	5'-GTTAAGCAGTACAGCCCCAAA-3'	5'-AGGGCATATCCAACAACAAACTT-3'
**hVAV1 shRNA sequence**		
CCGGCGTCGAGGTCAAGCACATTAACTCGAGTTAATGTGCTTGACCTCGACGTTTTTG		
**Primers for sequencing the mutants**		
Gene	Forward primer	Reverse primer
E59K	5'-TGGTGTCCTTCTGTGTCAGC-3'	5'-CATAGATCTCGTCGCCTTCC-3'
D517E	5'-ACACTGCGACAGATCACCAA-3'	5'-CAGCGGTAGCCCTGATAGAA-3'
L801P	5'-CCTCCTCAGGACCTGTCTGT-3'	5'-CCTTGCTGTCCCTTCTTGTT-3'
**Primers for PCR**		
Gene	Forward primer	Reverse primer
hVAV1	5'-TCTGCCCAAGATGGAGGTGTTTCA-3'	5'-TTCGTGAGCTCCACAATGTCTCCA-3
GPDH	5'-TCGACAGTCAGCCGCATCTTCTTT-3'	5'-ACCAAATCCGTTGACTCCGACCTT-3'
